# Risk factor analysis of insufficient fluid intake among urban adults in Wuxi, China: a classification and regression tree analysis

**DOI:** 10.1186/s12889-020-8380-y

**Published:** 2020-03-04

**Authors:** Hao Zheng, Juan Fei, Lan Zhang, Weijie Zhou, Zhen Ding, Wenbiao Hu

**Affiliations:** 1 Department of Environmental Health, Jiangsu Provincial Center for Disease Control and Prevention, No. 172 Jiangsu Road, Nanjing, 210009 China; 2 National Institute of Environmental Health, Chinese Center for Disease Control and Prevention, No. 29 Nanwei Road, Beijing, 100050 China; 3 Department of Public Health, Wuxi Center for Disease Control and Prevention, No. 499 Jincheng Road, Wuxi, 214023 China; 4 School of Public Health and Social Work, Institute of Health and Biomedical Innovation, Queensland University of Technology, Brisbane, Queensland 4059 Australia

**Keywords:** Fluid intake, Risk factors, Adults, CART

## Abstract

**Background:**

Dehydration due to insufficient fluid intake (IFI) is detrimental to health. This cross-sectional study aimed to assess the fluid intake of urban adults in Wuxi, China, and to identify potential risk factors contributing to IFI.

**Methods:**

Adults were selected from the urban area of Wuxi, China, using a multiple-stage random sampling method. The fluid intake information was obtained with a 24-h self-reported diary over seven consecutive days in both summer and winter of 2015. A classification and regression tree (CART) analysis was conducted to detect the potential risk factors associated with IFI. CART is a machine-learning algorithm that portions the data into subsets by threshold.

**Results:**

A total of 584 adults aged 18–87 years were included. The results showed that the median (P25–P75) values of daily fluid intake of the participants were 1100 (800–1550) mL in summer and 1000 (750–1300) mL in winter. Women had a higher prevalence of IFI than men in both summer (odds ratio (OR) = 2.683, 95% confidence interval (CI): 1.830–3.934) and winter (OR = 2.636, 95% CI: 1.677–4.142). The results of CART analysis showed that, in summer, BMI < 25 kg/m^2^ (probability: 64.2%) and age < 64 years (probability: 67.4%) were main risk factors of IFI for men, and BMI < 29 kg/m^2^ (probability: 81.6%) and living in C Community (probability: 86.7%) were main risk factors for women. In winter, age < 40 years (probability: 81.8%) and BMI < 20 kg/m^2^ (probability: 94.5%) were identified as main risk factors of IFI for men and women, respectively.

**Conclusions:**

Most of the participants living in the study site had IFI. The fluid consumption varied by gender, age, location, and BMI. The findings could be useful for the implementation and optimization of intervention programs by identifying the individuals who may at greater risk of dehydration.

## Background

Water is an essential constituent of the human body. It acts not only as a medium involved in reaction processes but also as a carrier transporting nutrients and wastes in and out of cells [1, 2]. Adequate fluid intake plays an important role in maintaining water balance, and dehydration may occur when the water balance is broken. It has been reported that dehydration is associated with urological, gastrointestinal, circulatory, and neurological disorders [3–6]. Even mild dehydration has an adverse impact on cognitive and performance functions, and elderly people are vulnerable to water deficit [6, 7].

Since the human body itself cannot produce enough water to sustain life, we mainly ingest water from plain water, beverages, and food moisture [8]. Fluid consumption requirements vary between individuals and are influenced by many factors, such as physical activity levels, environmental conditions, and physiological status [8]. The recommended daily adequate intake for total water intake set by the Food and Nutrition Board of the Institute of Medicine in the United States is 3.7 L and 2.7 L for young men and women, respectively [9]. The European Food Safety Authority set the threshold of adequate total water intake at 2.5 L/day for men and 2 L/day for women [10]. The World Health Organization recommends a fluid intake of 1.5 L/day [11]. In 2016, the Chinese Nutrition Society (CNS) recommended a daily adequate intake for adults ranging from 1.5 to 1.7 L [12]. Previous research in four Chinese cities showed that male adults had a higher fluid intake than females and significant differences were found for fluid intake among the four cities [13]. Similarly, a recent national survey performed by Zhang et al.in 27 cities in China, suggested that the fluid intake was significantly higher in male vs female, and a significant difference was found for fluid intake between cities with different socioeconomic status [14]. Some studies indicated that the water intake was significantly higher in boys than in girls among primary and middle school students [15, 16], and was higher in urban than in rural regions [17, 18]. In addition, a few studies suggested that drinking behaviors (different consumption periods and times) had a close association with water consumption [16, 19, 20]. Zhang et al. identified 24-h urine volume and osmolality as possible key predictors for hydration status among male college students [21]. Wang et al. reported that obese adolescents drank more water than normal weight and overweight counterparts in Shanghai, China [22]. Moreover, it has been reported that the amount of water intake was affected by season, gender, and body mass index (BMI) in China [23–25]. These findings suggest that the fluid intake may differ by region and that potential risk factors contributing to IFI need to be further explored.

Understanding the interaction of determinants of IFI can facilitate the planning and implementation of prevention or intervention programs. However, most previous studies used only univariate or multivariate models, which did not assess the interaction across variables [13, 14, 26–28]. The classification and regression tree (CART) analysis is a very useful method that can potentially better accommodate these complex interactions since they avoid some of the assumptions associated with linear regression [29]. In the present study, we aimed to assess the situation of fluid intake among adults in the urban area of Wuxi, China and to identify potential risk factors contributing to IFI using CART analysis.

## Methods

### Study area

This study was conducted in Wuxi, which is a modernized city located in Southeast Jiangsu Province, with geographical coordinates between 31.70 to 32.00 N and 119.31 to 120.36 E. In 2015, Wuxi had a total area of 4628 km^2^ and a resident population of 6.5 million, and the urban population accounted for 51.7% of the total population. People aged 0–14, 15–64, and ≥ 65 years accounted for 10.4, 78.2, and 11.4% of the total population, respectively. The average annual temperature was 16.8 °C and ranged from − 5.3 to 38.5 °C. The male/female sex ratio was 0.98. The GDP of Wuxi was 850 billion RMB (125 billion US dollars) in 2015. All the information above was derived from the Statistical Yearbook of Wuxi in 2015 [30].

### Sample size and sampling design

The sample size was calculated according to the formula provided by Lwanga and Lemeshow [31], assuming that the prevalence of IFI was 32%, as reported among adults in four Chinese cities [13]. According to the OpenEpi calculation procedure (OpenEpi, Version 3.01, Atlanta, USA), with confidence level set at 95%, power set at 80, 5% for confidence limits, and 1.7 for design effect, the sample size required was 569. Taking no response into account, the number of adults for the study was estimated as 600.

**Fig. 1:**
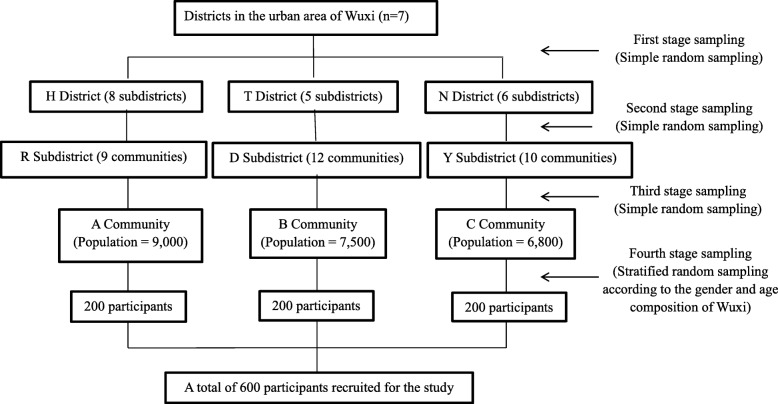
Description of multiple-stage random sampling method in the study

In our study, a multiple-stage random sampling method was used to select the study population of the urban area. In general, urban areas of cities in China have three classes of administrative divisions: district, subdistrict, and community. There were seven districts, 41 subdistricts and 442 communities in the urban area of Wuxi in 2015. First, three districts were randomly selected from all seven districts. Then, one subdistrict was randomly selected from each selected district. Finally, one community was randomly selected from each selected subdistrict, and 200 adults from each community were randomly selected. Therefore, a total of 600 adults from three communities that were representative of the urban population of Wuxi were recruited for the study. Details of the sampling method are presented in Fig. 1.

The inclusion criteria: healthy subjects aged ≥18 years old were included.

The exclusion criteria: subjects were excluded if they were diagnosed with kidney, liver, heart, or endocrine diseases that may influence fluid consumption.

### Data collection

Before the beginning of the household survey, all participants were informed of the aims and procedures of the survey, and those who signed their informed consent were recruited. Then, each participant was instructed to complete a 24-h self-reported fluid intake diary. The participants recorded the types and amounts of fluid consumed over seven consecutive days (starting on the day of the survey). A graduated container (100 mL) was provided to each participant for accurate measurement of their fluid consumption. In addition, the demographic information of the participants, including age, gender, residential address, ethnicity, occupation, height, and weight, were collected by the investigators. Details about the fluid intake questionnaire are presented in Additional file 1.

We performed two rounds of the survey on the same participants in summer (August) and winter (December) in 2015. Outdoor temperature and humidity data of the study site for the seven-day study period were collected from the Meteorological Bureau of Wuxi. To increase the response rate, we chose the weekend as the first day of the household survey.

### Variables

In our study, the volume of fluid intake was the sum of the following two sources: (a) plain water, including tap water, mineral water, purified water and tea water and (b) beverages, including tea drinks, carbonated drinks, fruit and vegetable drinks, plant protein drinks, energy drinks, solid drinks, plant drinks, milk drinks, coffee, milk and yogurt.

The CNS recommended a daily adequate intake of 1.5–1.7 L among adults who perform light physical activity under a moderate environmental temperature in 2016. Therefore, a daily fluid intake of less than 1.5 L was defined as IFI in our study.

Height in meters (m) and weight in kilograms (kg) were measured following a standardized procedure with steel tapes (DL3796, Deli, Zhejiang, China) and portable scales (EB9005L, Xiangshan, Guangdong, China) by investigators. Height and weight were measured twice (once in the summer and once in the winter), and average height and weight for each participant were calculated. The BMI was calculated as weight/height squared (kg/m^2^) and classified into four levels: < 18.5 kg/m^2^ (underweight), 18.5–23.9 kg/m^2^ (normal weight), 24–27.9 kg/m^2^ (overweight), and ≥ 28 kg/m^2^ (obesity) [32].

The participants who were mainly engaged in physical activity (blue collar) were classified as labor workers, and the rest were classified as nonlabor workers.

### Statistical analysis

The amount of fluid intake was presented as the median with 25th and 75th percentiles (P25–P75). Comparisons of the amount of fluid intake by demographic information, such as season, age, gender, location, ethnicity, occupation, BMI were performed with nonparametric test.

To identify the potential risk factors related to IFI (i.e., having IFI or not), the demographic variables were considered individually as independent variables included in Pearson’s chi-square test. Then, multivariate analysis was applied to confirm the risk factors by logistic regression analysis (Enter), and the results were presented as adjusted odds ratio (OR) with 95% confidence interval (CI). Factors with *p* <  0.1 in Pearson’s chi-square test were selected as the independent variables in the logistic regression.

CART is a machine-learning algorithm that portions the data into subsets by threshold. A CART analysis model was applied to further explore the association between IFI and demographic variables. The analysis was performed for men and women in different seasons.

Descriptive analysis, nonparametric test, Pearson’s chi-square test, and multivariate analysis were performed using SPSS software (version 20.0; IBM SPSS Institute, Inc., Armonk, NY, USA). Regression tree analysis was performed using R software (version 3.4.0, Package: rpart). Statistical significance was set at *p* <  0.05. All data were checked for completeness and accuracy before analyses. Incomplete or no-response diaries were excluded from the final analyses.

## Results

### Characteristics of the participants

In total, 2.7% (16/600) of the participants were excluded, and 584 adults aged 18–87 years from three communities (A, B, and C) were included in the study. The participants were categorized into three age groups: 18–34 years (*n* = 195, 33.4%), 35–54 years (*n* = 191, 32.7%), and ≥ 55 years (*n* = 198, 33.9%). The average age of the participants was 45.0 ± 16.9 years.

Among the participants, 278 (47.6%) were men and 306 (52.4%) were women, with a male/female sex ratio of 0.908. The majority of the participants were Han Chinese (*n* = 579, 99.1%) and claimed to be nonlabor workers (*n* = 482, 82.5%). In addition, individuals classified as BMI < 18.5 kg/m^2^ and BMI ≥ 28 kg/m^2^ accounted for 4.8% (*n* = 28) and 4.3% (*n* = 25), respectively. The general characteristics of the participants in the study are described in Table 1.

**Table 1:** Characteristics of the participants in the study

Variables	No. of Participants	Percent
Total	584	100
Gender		
Man	278	47.6
Woman	306	52.4
Age (years)		
18–34	195	33.4
35–54	191	32.7
≥ 55	198	33.9
Location (Community)		
A	193	33.0
B	192	32.9
C	199	34.1
Ethnicity		
Han	579	99.1
Others	5	0.9
Labor worker		
Yes	102	17.5
No	482	82.5
BMI (kg/m^2^)		
< 18.5	28	4.8
18.5–23.9	368	63.0
24–27.9	163	27.9
≥ 28	25	4.3

### Temperature and humidity

The mean values of outdoor temperature and humidity for the seven days were 28.3 °C and 74.4% in summer and 5.9 °C and 78.6% in winter. Details of the outdoor temperature and humidity of the seven days are presented in Additional file 2.

### Daily fluid intake of the participants

The median (P25–P75) daily fluid intake of the participants was 1050 (750–1400) mL. Regarding the different seasons, the median (P25–P75) daily fluid intake of the participants was 1000 (750–1300) mL in winter, which was significantly lower than that in summer (1100 (800–1550) mL, *p* <  0.0001). For men, the daily fluid intake was significantly higher in summer than in winter (1300 vs 1100 mL/day, *p* <  0.0001). However, no significant difference was found for women between summer and winter.

Women were more likely to consume less fluids than men in both summer (1000 vs 1300 mL/day, *p* <  0.0001) and winter (996 vs 1100 mL/day, *p* <  0.0001). Individuals with BMI < 18.5 kg/m^2^ were more likely to consume less fluids than individuals with BMI 24–27.9 kg/m^2^ in summer (800 vs 1300 mL/day, *p* = 0.041). A significant difference in fluid consumption was observed in different communities in winter (*p* = 0.002): individuals living in A and B Community had a lower consumption of fluids than those in C Community (1000 vs 1100 mL/day, *p* = 0.029; 950 mL vs 1100 mL/day, *p* = 0.002). However, there was no significant difference among the communities in summer. The daily fluid intake of certain adults in summer and winter is shown in Table 2.

**Table 2:** Description of daily fluid intake (mL) among adults (18–87 years) in summer and winter (*n* = 584)

Variables	N	Summer		Winter	
		Median (P25–P75)	*p*	Median(P25–P75)	*p*
Gender					
Man	278	1300 (900–1750)	< 0.0001	1100 (800–1450)	< 0.0001
Woman	306	1000 (700–1350)		996 (700–1250)	
Age (years)					
18–34	195	1050 (700–1550)	0.062	950 (700–1250)	0.128
35–54	191	1200 (800–1500)		1000 (800–1400)	
≥ 55	198	1200 (819–1550)		1025 (800–1300)	
Location (Community)					
A	193	1220 (850–1600)	0.206	1000 (750–1300)*	0.002
B	192	1100 (758–1600)		950 (650–1250)**	
C	199	1100 (700–1430)		1100 (900–1300)	
Labor worker					
Yes	102	1100 (650–1450)	0.094	923 (650–1200)	0.025
No	482	1110 (800–1550)		1000 (750–1300)	
BMI (kg/m^2^)					
< 18.5	28	800 (475–1339)***	0.012	900 (613–1338)	0.374
18.5–23.9	368	1100 (750–1500)		1000 (750–1300)	
24–27.9	163	1300 (850–1650)		1000 (750–1250)	
≥ 28	25	1200 (926–1650)		900 (700–1150)	

*: *p* = 0.029, compared with C Community; **: *p* = 0.002, compared with C Community; ***: *p* = 0.041, compared with BMI 24–27.9 kg/m^2^

### Prevalence and risk factors associated with IFI using multivariate logistic regression

In total, 898 out of 1168 (76.9%) participants had IFI. In winter, the prevalence of IFI was 82.5%, which was significantly higher than that in summer (71.2%) (OR = 1.908, 95% CI: 1.444–2.522). For men, the prevalence of IFI was significantly higher in winter than in summer (75.5% vs 60.4%, OR = 2.022, 95% CI: 1.405–2.910). However, no significant difference was found for women between summer and winter.

The results of multivariate logistic regression suggested that women had a higher prevalence of IFI than men in both summer (81.0 vs 60.4%, OR = 2.683, 95% CI: 1.830–3.934) and winter (88.9 vs 75.5%, OR = 2.636, 95% CI: 1.677–4.142). The prevalence of IFI among individuals aged 35–54 years was significantly lower than that among those aged 18–34 years in winter (77.5 vs 86.2%, OR = 0.534, 95% CI: 0.311–0.915). In addition, significant differences in fluid intake were noted in different communities. Individuals who lived in C Community were more likely to have IFI than those who lived in A Community in summer (77.9 vs 65.3%, OR = 1.921, 95% CI: 1.208–3.056). However, the prevalence of IFI did not differ across occupations or BMI groups in either summer or winter. Table 3 presents the results of the multivariate analysis that identified the risk factors of IFI in summer and winter.

**Table 3:** Multivariate analysis of risk factors associated with IFI among adults (18–87 years) in summer and winter (*n* = 584)

Variables		Summer			Winter		
	N	No. (%)	aOR (95% CI)	*p*	No. (%)	aOR (95% CI)	*p*
Gender							
Woman	306	248 (81.0)	2.683 (1.830–3.934)	< 0.001	272 (88.9)	2.636 (1.677–4.142)	< 0.001
Man	278	168 (60.4)			210 (75.5)		
Age							
18–34	195	139 (71.3)			168 (86.2)	1	0.056
35–54	191	140 (73.3)			148 (77.5)	0.534 (0.311–0.915)	0.022
≥ 55	198	137 (69.2)			166 (83.8)	0.828 (0.471–1.453)	0.510
Location (Community)							
A	193	126 (65.3)	1	0.022	155 (80.3)		
B	192	135 (70.3)	1.265 (0.810–1.975)	0.302	160 (83.3)		
C	199	155 (77.9)	1.921 (1.208–3.056)	0.006	167 (83.9)		
Labor worker							
Yes	102	79 (77.5)			88 (86.3)		
No	482	337 (69.9)			394 (81.7)		
BMI (kg/m^2^)							
< 18.5	28	22 (78.6)			25 (89.3)		
18.5–23.9	368	275 (74.7)			301 (81.8)		
24–27.9	163	105 (64.4)			135 (82.8)		
≥ 28	25	14 (56.0)			21 (84.0)		

### Risk factors of IFI using CART analysis

**Fig. 2:**
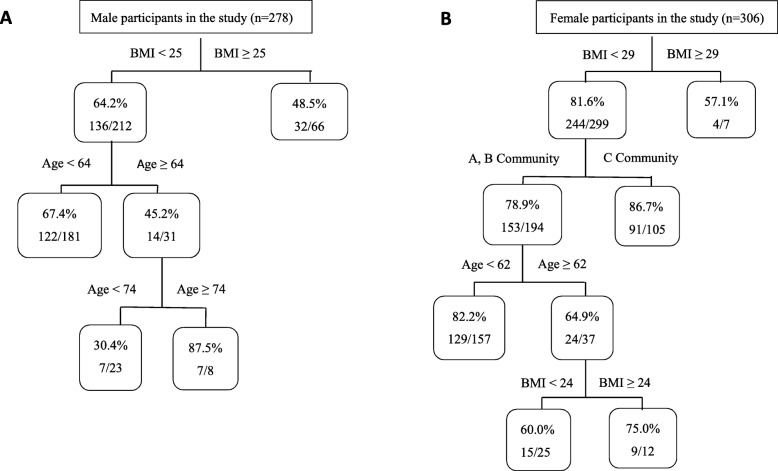
Risk factors contributing to IFI among adults (18–87 years) in summer according to CART analysis in the study (age: years, BMI: kg/m^2^). The figures in the rectangle indicate the probability of IFI. **a**: Male, **b**: Female

The risk factors contributing to IFI in summer according to CART analysis are delineated in Fig. 2. The prevalence of IFI in summer was 60.4% (168/278) for men and 81.0% (248/306) for women. For the first classified factor, men with BMI < 25 kg/m^2^ (64.2%, 136/212) were at greater risk of having IFI than those with BMI ≥ 25 kg/m^2^ (48.5%, 32/66). Moreover, for men with BMI < 25 kg/m^2^, the probability of IFI was higher among those aged < 64 years (67.4%, 122/181) than those aged ≥64 years (45.2%, 14/31) (Fig. 2a).

For women, BMI < 29 kg/m^2^ (81.6%, 244/299) was identified as the first risk factor for IFI in summer. In addition, individuals with BMI < 29 kg/m^2^ who lived in C Community (86.7%, 91/105) were more likely to have IFI than those who lived in A and B Communities (78.9%, 153/194). Furthermore, individuals with BMI < 29 kg/m^2^ in A and B Communities aged < 62 years (82.2%, 129/157) had a higher probability than those aged ≥62 years (64.9%, 24/37) (Fig. 2b).

**Fig. 3:**
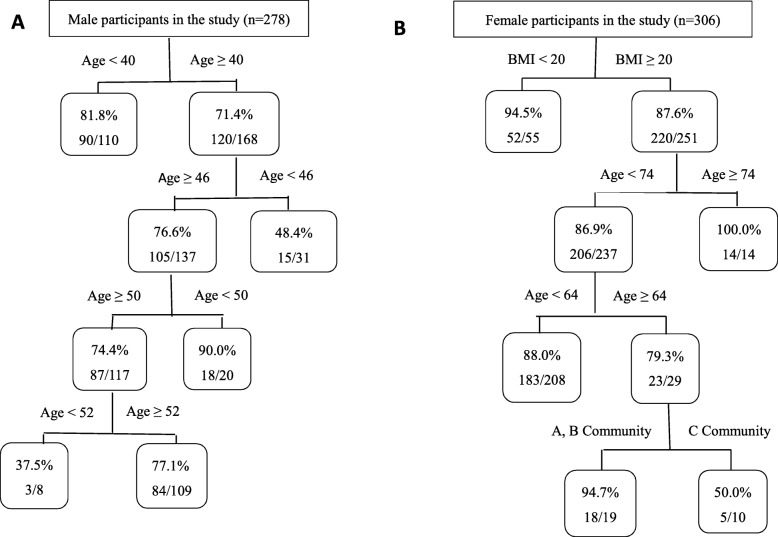
Risk factors contributing to IFI among adults (18–87 years) in winter according to CART analysis in the study (age: years, BMI: kg/m^2^). The figures in the rectangle indicate the probability of IFI. **a**: Male, **b**: Female

The risk factors contributing to IFI in winter according to CART analysis are delineated in Fig. 3. The probability of IFI in winter was 75.5% (210/278) for men and 88.9% (272/306) for women. The figure shows that men aged < 40 years (81.8%, 90/110) had a higher probability of IFI than those aged ≥40 years (71.4%, 120/168) in winter. Furthermore, the probability of IFI was higher among men aged ≥46 years (76.6%, 105/137) than among those aged ranging from ≥40 to < 46 years (48.4%, 15/31) (Fig. 3a).

For women in winter, a BMI of 20 kg/m^2^ was recognized as the first threshold, with individuals with BMI < 20 kg/m^2^ (94.5%, 52/55) having a higher probability of IFI than those with BMI ≥ 20 kg/m^2^ (87.6%, 220/251). Furthermore, for women with BMI ≥ 20 kg/m^2^, the prevalence of IFI was higher among those aged ≥74 years (100.0%, 14/14) than among those aged < 74 years (86.9%, 206/237). Additionally, for women with BMI ≥ 20 kg/m^2^ and aged < 74 years, the probability of IFI was higher among those aged < 64 years (88.0%, 183/208) than among those aged ≥64 years (79.3%, 23/29) (Fig. 3b).

## Discussion

This study assessed the fluid consumption among adults using a 24-h self-reported diary over seven consecutive days based on the urban population of Wuxi, China. Since plain water and beverages account for approximately 80% of the total water inputs, this study focused on the sum of plain water and beverages [10]. Our results suggested that most of the participants aged 18–87 years had IFI and the fluid consumption varied by gender, age, location, and BMI. In winter, the age of < 40 years (probability: 81.8%) and BMI < 20 kg/m^2^ (probability: 94.5%) were identified as main risk factors contributing to IFI for men and women, respectively. Moreover, both the high-order interactive effects and thresholds of risk factors related to IFI have been detected in our study.

The results showed that the median daily fluid intake was 1050 mL, which was lower than the findings of a survey of adults aged 18–55 years in 27 Chinese cities (1214 mL) [14] and a survey among young adults aged 18–23 years in Baoding, China (1135 mL) [33]. These differences may be due to the different climate conditions and characteristics of the study populations. In addition, residents of Wuxi tended to eat porridge in the morning and evening, and the water intake from the porridge may have compensated for a portion of the total water intake. Ma et al. reported that the median daily fluid intake among 1483 adults aged 18–60 years in China was 1488 mL, which is higher than the findings of our study [13]. However, the Ma et al. study was performed in Beijing, Shanghai, Chengdu, and Guangzhou. These four cities have a higher socioeconomic status than Wuxi, which may potentially increase the fluid intake of the participants [34]. The results also showed that most adults (76.9%) failed to meet the recommended adequate intake guidelines set by CNS, a finding similar to that of the survey performed by Zhang N et al. (72%) [14].

Our results also indicated that women consumed significantly less fluids than men in both summer and winter. Men generally have a higher metabolic rate than women at the same age due to the physiological disparity, and hence, the fluid requirements for men are higher than those for women. Several studies in China have reported that males had a higher fluid consumption than females among primary, middle school students [16–18], college students [23, 33], and adults [13, 14]. Our result is in accordance with these findings of China. Surveys performed in Ireland and the United Kingdom also reported that male adults consumed more fluids than females [35, 36]. However, studies conducted in Mexico and Spain reported that female adults had more fluid consumption than males [37, 38]. The differences may be due to the different drinking habits and age groups of the study population. Several studies have reported that the fluid intake of Chinese was affected by season [23–25]. However, our study found that men had a higher prevalence of IFI in winter than in summer, which is different from the fluid intake of women. This result suggests that the fluid intake of men is more prone to be affected by season than women.

The results of CART analysis suggested that age < 40 years was the first risk factor for men in winter. Multivariate analysis also indicated that the prevalence of IFI was significantly higher among those aged 18–34 years than among those aged 35–55 years. We speculated that men aged < 40 years are usually engaged in high-level activity and therefore may require more fluids. Additionally, young adults tend to ignore rehydration. Therefore, male adults aged < 40 years were considered as a vulnerable group to IFI in winter. The difference in fluid intakes across different age groups was also reported in other studies [26, 28, 39].

The results of CART analysis suggested that BMI < 25 kg/m^2^ and age < 64 years were the first and second risk factors of IFI for men in summer, respectively, and BMI < 20 kg/m^2^ was the first risk factor for women in winter. We observed that the individuals with BMI < 18.5 kg/m^2^ had a higher prevalence of IFI than individuals in other BMI groups in both summer and winter, although no statistical difference was found in the present study. Individuals with low BMI might have a lower level of water loss than those with high BMI. However, due to the limitation of the cross-sectional study design, no causal relations between low BMI and IFI in the study can be determined. A survey conducted among Irish adults indicated an association between lower fluid intake and higher BMI [35]. Instead, Kant et al. found an association between higher body mass and higher water intake in the adult US population [40]. Furthermore, data also suggested that age < 64 years was associated with IFI for men in our study. Since the age of retirement for men is between 60 and 65 years in China, the retired population may have more spare time to pay attention to maintaining a healthy body hydration status. In contrast, S.M. Roche et al observed that men at an older age had lower water consumption in Canadian communities [26]. This association was also reported by other studies [41–44].

The volume of fluid intake varies across countries around the world. A systematic review was conducted to assess fluid intake from 2000 to 2013 and reported that the daily fluid intake among adults was 0.8–3.4 L worldwide [39]. Another survey conducted in 15 countries also showed considerable variation in fluid intake across countries [45]. In our study, the volume of fluid intake varied significantly across different communities. The CART analysis showed that, in summer, participants who lived in C Community had IFI, especially women with BMI < 29 kg/m^2^. Conversely, we also found that the volume of fluid intake in C Community was significantly higher than that in A and B Communities in winter. One possible reason is that the activity patterns of individuals in C Community may differ in summer and winter. It is also noteworthy that C Community was a relatively older community with a lower socioeconomic status than A and B Communities [30]. Studies from the United States and the United Kingdom reported that lower-income adults may be at greater risk of inadequate hydration than higher-income adults [34, 43, 46]. Zhang et al. also found that low socioeconomic status may decrease fluid consumption [14].

To the best of our knowledge, this is the first cross-sectional study using CART analysis to determine the high-order interactions and thresholds of risk factors associated with IFI. Furthermore, risk factors were ranked according to their importance to IFI. The detection of risk factors can be useful for the identification of individuals who may at greater risk of dehydration. The seven-day self-reported method for assessing fluid intake has been validated for accuracy and reliability [47, 48], and this method has been widely used in previous studies [13, 14, 33, 45].

Limitations should be mentioned for our study. First, several factors that may potentially affect the fluid intake, such as season (spring and autumn), physical activity levels, income, and educational levels of the participants, were not considered. Second, according to the daily adequate fluid intake (1.5–1.7 L) recommended by CNS, 1.5 L was used as the threshold of IFI for general population in the present study. Detailed analysis using gender/age differentiated threshold of IFI should be employed in the further study. Finally, our study was conducted among the urban population in Wuxi, so the results may not be generalizable to the whole population. More extensive and large-scale investigations are required in the future.

## Conclusions

Most of the participants living in the study site had IFI. The fluid consumption varied by gender, age, location, and BMI. The findings could be useful for the implementation and optimization of intervention programs by identifying the individuals who may at greater risk of dehydration.

## Supplementary information

**Additional file 1:** Details of the fluid intake questionnaire in the study.

**Additional file 2:** The temperature and humidity of seven days.

## Data Availability

The datasets used during the current study are available from the corresponding authors on reasonable request.

## Abbreviations

BMI: Body mass index
CART: Classification and regression tree
CI: Confidence interval
CNS: Chinese nutrition society
IFI: Insufficient fluid intake
OR: Odds ratio
