# Supplementary material for: Risk factor analysis of insufficient fluid intake among urban adults in Wuxi, China: a classification and regression tree analysis
Source: BMC Public Health. 2020 Mar 4;20:286. doi: 10.1186/s12889-020-8380-y (PMC7057576; doi:10.1186/s12889-020-8380-y)
Supplement: Supplementary file 1 — Additional file 1: Details of the fluid intake questionnaire in the study. [file 12889_2020_8380_MOESM1_ESM.docx]

**Additional file 1:** Details of the fluid intake diary in the study

1. Basic information

1. Name:
2. Phone:
3. Address:
4. Researcher Name1:
5. Researcher Name2:
6. Date:
7. Coding:

2. Demographic information

1. Your birthday: Year Month
2. Gender: 1. Male; 2. Female
3. Ethnicity: 1. Han; 2. Others
4. Labor worker: 1. Yes; 2. No
5. Height: cm; Weight: kg

3. Are you feeling ill today? 1=no, 2=yes (if yes, please describe the symptoms)

| **Fluid intake** | | | | |
| --- | --- | --- | --- | --- |
| Morning | **Occasions** | **NO.** | **Type of fluids** | **Volume (mL)** |
|  | Before breakfast | B | Plain water^a^ |  |
|  |  | D | Beverages^b^ |  |
|  | Breakfast | B | Plain water |  |
|  |  | D | Beverages |  |
|  | Between breakfast and lunch | B | Plain water |  |
|  |  | D | Beverages |  |
| Afternoon | Lunch | B | Plain water |  |
|  |  | D | Beverages |  |
|  | Between lunch and supper | B | Plain water |  |
|  |  | D | Beverages |  |
| Evening | Supper | B | Plain water |  |
|  |  | D | Beverages |  |
|  | After supper | B | Plain water |  |
|  |  | D | Beverages |  |
|  | Night | B | Plain water |  |
|  |  | D | Beverages |  |

a: Plain water, including tap water, mineral water, purified water and tea water.

b: Beverage, including tea drinks, carbonated drinks, fruit and vegetable drinks, plant protein drinks, energy drinks, solid drinks, plant drinks, milk drinks, coffee, milk and yogurt.
