# Supplementary material for: Risk factor analysis of insufficient fluid intake among urban adults in Wuxi, China: a classification and regression tree analysis
Source: BMC Public Health. 2020 Mar 4;20:286. doi: 10.1186/s12889-020-8380-y (PMC7057576; doi:10.1186/s12889-020-8380-y)
Supplement: Supplementary file 2 — Additional file 2: The temperature and humidity of seven days. [file 12889_2020_8380_MOESM2_ESM.docx]

**Additional file 2:**

The temperature and humidity of the seven days.

|  | Summer | |  | Winter | |
| --- | --- | --- | --- | --- | --- |
|  | Temperature (℃) | Humidity (%) |  | Temperature (℃) | Humidity (%) |
| Day 1 | 31 | 73 |  | 6 | 53 |
| Day 2 | 29.5 | 70 |  | 9 | 72 |
| Day 3 | 29 | 77 |  | 7 | 77 |
| Day 4 | 27.5 | 82 |  | 4.5 | 87 |
| Day 5 | 26.5 | 71 |  | 5 | 95 |
| Day 6 | 27 | 78 |  | 5.5 | 89 |
| Day 7 | 27.5 | 70 |  | 4.5 | 77 |
